# Development of a Video-Observed Therapy System to Improve Monitoring of Tuberculosis Treatment in Thailand: Mixed-Methods Study

**DOI:** 10.2196/29463

**Published:** 2021-07-27

**Authors:** Ponlagrit Kumwichar, Virasakdi Chongsuvivatwong, Tagoon Prappre

**Affiliations:** 1 Epidemiology Unit Faculty of Medicine Prince of Songkla University Songkhla Thailand

**Keywords:** app, mixed-methods analysis, remote monitoring, therapy, tuberculosis, user experience, video directly observed therapy, video-enhanced therapy, video-observed therapy

## Abstract

**Background:**

Directly observed therapy programs for monitoring tuberculosis (TB) treatment in Thailand are unsustainable, especially during the COVID-19 pandemic. The current video-observed therapy (VOT) system, the Thai VOT (TH VOT), was developed to replace the directly observed therapy program.

**Objective:**

This study aimed to describe the VOT system design and identify the potential for system improvements.

**Methods:**

This pilot study was conducted in Na Yong district, a small district in Trang province, south of Thailand. The TH VOT system consists of a smartphone app for patients, a secured web-based platform for staff, items used, and standard operating procedures. There were three groups of users: observers who were TB staff, healthy volunteers as simulated patients, and patients with active TB. All participants were trained to follow the standard operating procedures. After 2-week usage, VOT session records were analyzed to measure the compliance of the patients and observers. The User Experience Questionnaire was used to lead the participant users to focus on 6 standard dimensions of usability, and was supplemented with an in-depth interview to identify potential system improvements from users’ experience.

**Results:**

Only 2 of 16 patients with currently active TB had a usable smartphone. Sixty of 70 drug-taking sessions among 2 patients and 3 simulated patients in 2 weeks were recorded and uploaded. Only 37 sessions were inspected by the observers within 24 hours. All participants needed a proper notification system. An audit system was also requested.

**Conclusions:**

Before upscaling, the cost of smartphone lending, audit management, and notification systems should be elucidated.

## Introduction

Thailand is currently one of 30 countries worldwide with the highest tuberculosis (TB) rate [1]. Directly observed treatment (DOT) has been implemented since 1996 [2]. Despite 2 previous studies showing the poor sustainability of DOT, the country has not made any change owing to a lack of alternative strategies [3,4]. As such, the National Tuberculosis Control Program Guideline has recommended health personnel as the first-choice observer [5]. However, family-based DOT is administered to 60%-75% of TB cases owing to the complacency of the health care system [6,7]. Family-based DOT is also complacent because of the nature of family relationships [8-10]. Ultimately, nobody has been formally accountable for DOT with regard to the patient. Consequently, the cumulative number of drug-resistant TB cases detected has increased from approximately 500 in 2014 to 1200 cases in 2019, which indicates the poor quality of DOT in the health care system [1]. Additionally, the health care situation has been worsened by the ongoing COVID-19 pandemic [11,12]. Therefore, newer studies are needed to develop a new observed therapy method to mitigate the existing issues and accelerate the End TB Strategy of the World Health Organization [1].

Recently, with advancements in smartphone technology, internet penetration has increased the access of the whole population to mobile phones and other electronic devices. Consequently, a new technology called video-observed therapy (VOT) has been introduced to replace DOT [13]. VOT is a platform that allows health personnel to observe medical ingestion through a television system. There are two types of VOT: synchronous VOT (S-VOT) and asynchronous VOT (A-VOT). S-VOT is the live form of VOT in which the patient and health care personnel interact in real time. However, A-VOT is a platform on which the patient records and uploads a video to the health service, and the health care personnel review the video later [14]. Two randomized controlled trials in the United Kingdom and the United States reported that both S-VOT and A-VOT could lower health service costs compared to traditional DOT [15,16].

In Thailand, the “TH VOT” has been developed by our group. This is an A-VOT system with a smartphone app available on the Google Play Store. A-VOT has been selected because the internet bandwidth in rural areas where patients with TB live is still too deficient to allow for S-VOT on a real-time interface. In the background, a secured website platform was developed, which allowed only the approved HCP to review the video. With standard operating procedures (SOPs), the TH VOT platform was tested in Na Yong district, Trang province, southern Thailand. The objectives of this study were to describe how the VOT system was designed and to identify potential system improvements.

## Methods

### Overview of the Study and the TH VOT System

In Thailand, a patient with active TB would be transferred to be monitored by the DOT program of a primary care unit (PCU) close to the patient’s home. Normally, 1 of the health care personnel at the PCU is assigned to be an observer to monitor medication adherence of the patient; this person is called the “TB staff.” The TB staff could also assign his/her observation to a local village health volunteer or a family member owing to the inadequate labor force. The TH VOT system was specifically designed to replace the DOT program in the setting. In the VOT system, an observer must be a PCU staff member. This study consisted of two parts: the system design and identification of potential system improvements.

### System Design

The TH VOT system included a smartphone app for patients, a secure web-based platform for staff, items used, and SOPs.

### Smartphone App for Patients

The TH VOT app was programmed using Dart language and had the following features:

1. Login interface: the first page that requires a username and password to sync with our online Structured Query Language database.
2. Main interface: only 1 main interface of this app with an obvious button to turn on the camera.
3. Video recorder: using a smartphone-based camera feature to record a video.
4. Uploading feature: a back-end function to compress the size of the recorded video to accelerate the uploading process, considering the low internet speed in Thailand.
5. Notification system: push notification that pops up on a screen at any time set by the user.

### Secure Website Platform for VOT Observers

**Figure 1:**
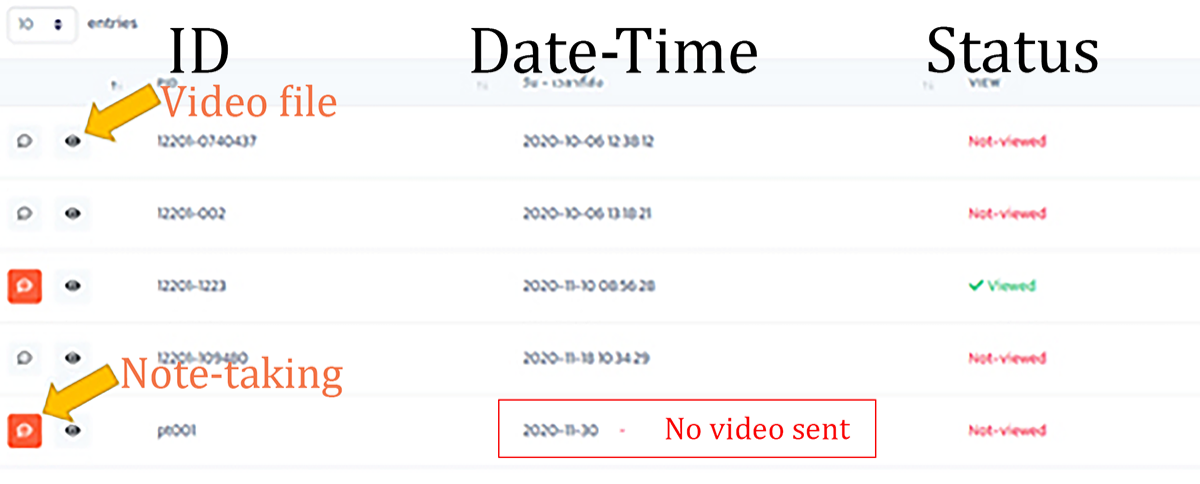
The review dashboard.

**Figure 2:**
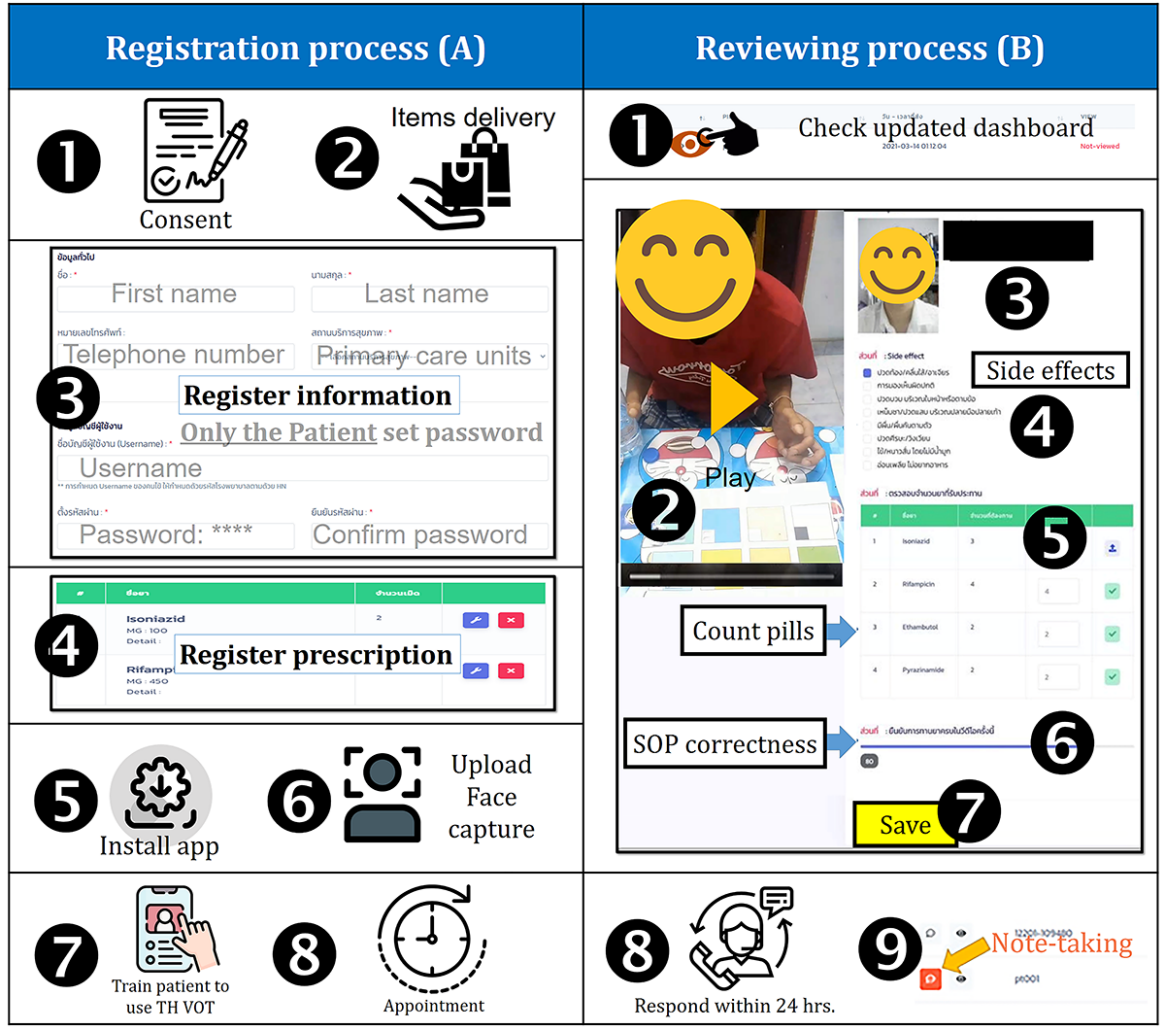
Standard operation procedures of the video-observed therapy at the patients' end. SOP: standard operating procedure, TH VOT: Thai video-observed therapy.

The website interface was coded using Cascading Style Sheets and its features were programmed in JavaScript:

1. Login interface: the first page that requires a username and password to sync with the same Structured Query Language database as that of the TH VOT app.
2. Main interface: an interface of the platform to choose 1 of 3 menus—a review dashboard, a registration page, and a user settings page.
3. Review dashboard: a daily automatically updated spreadsheet with records of registered patients in rows and information including note-taking, the video file with a button for pop-up video verification, patient identification number, time when the video arrived, and approval status in columns (Figure 1).
4. Registration: a webpage to register a patient under the direct responsibility of the observer (Figure 2A).
5. Video verification: a window pop-up after pressing a play button on the dashboard. The video file can be inspected at various speeds to be approved (Figure 2B).
6. User settings: a page to edit user information and choose an option of notification.
7. Notification system: LINE application programming interface that pushes the notification to the observer when the video has already arrived. LINE token access from the observer must be activated to use this feature.

In this pilot study, the notification system was omitted owing to budget-related issues.

### Items Used

**Figure 3:**
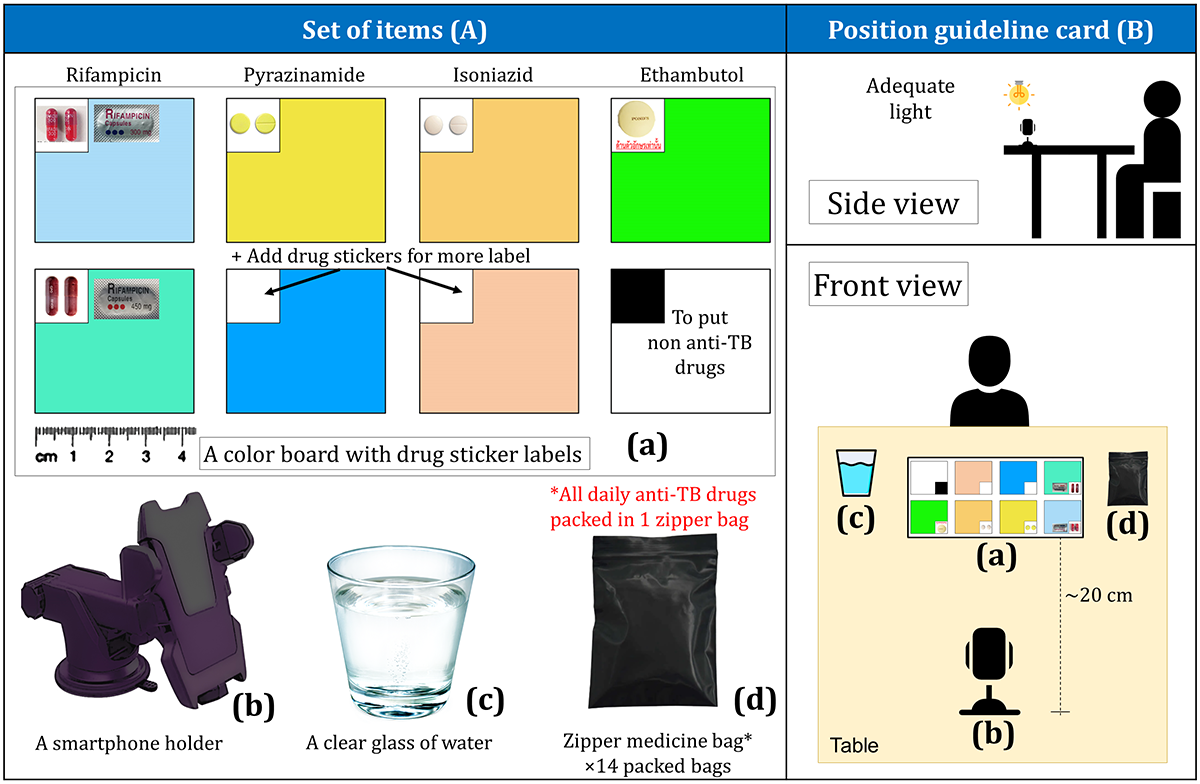
A set of items provided to a patient (A) and a position guideline for a patient (B). TB: tuberculosis.

Essential items that must be provided to patients are listed in Textbox 1 and illustrated in Figure 3.

**A list of items delivered to the patients.**

**Items:**

- A color board with drug labels (20×10 cm) (Figure 3A)
- A smartphone holder (Figure 3A).
- A clear glass (200 mL) (Figure 3A)
- 14 zipper medicine bags with a daily anti-TB dose packed inside (2 vitamin tablets per bag for a simulated patient) (Figure 3A).
- A position guideline for the patient (Figure 3B)

### SOPs

SOPs were divided into two series: patient SOP and observer SOP.

#### SOPs of the VOT at the Patients’ End (Patient SOP)

**Figure 4:**
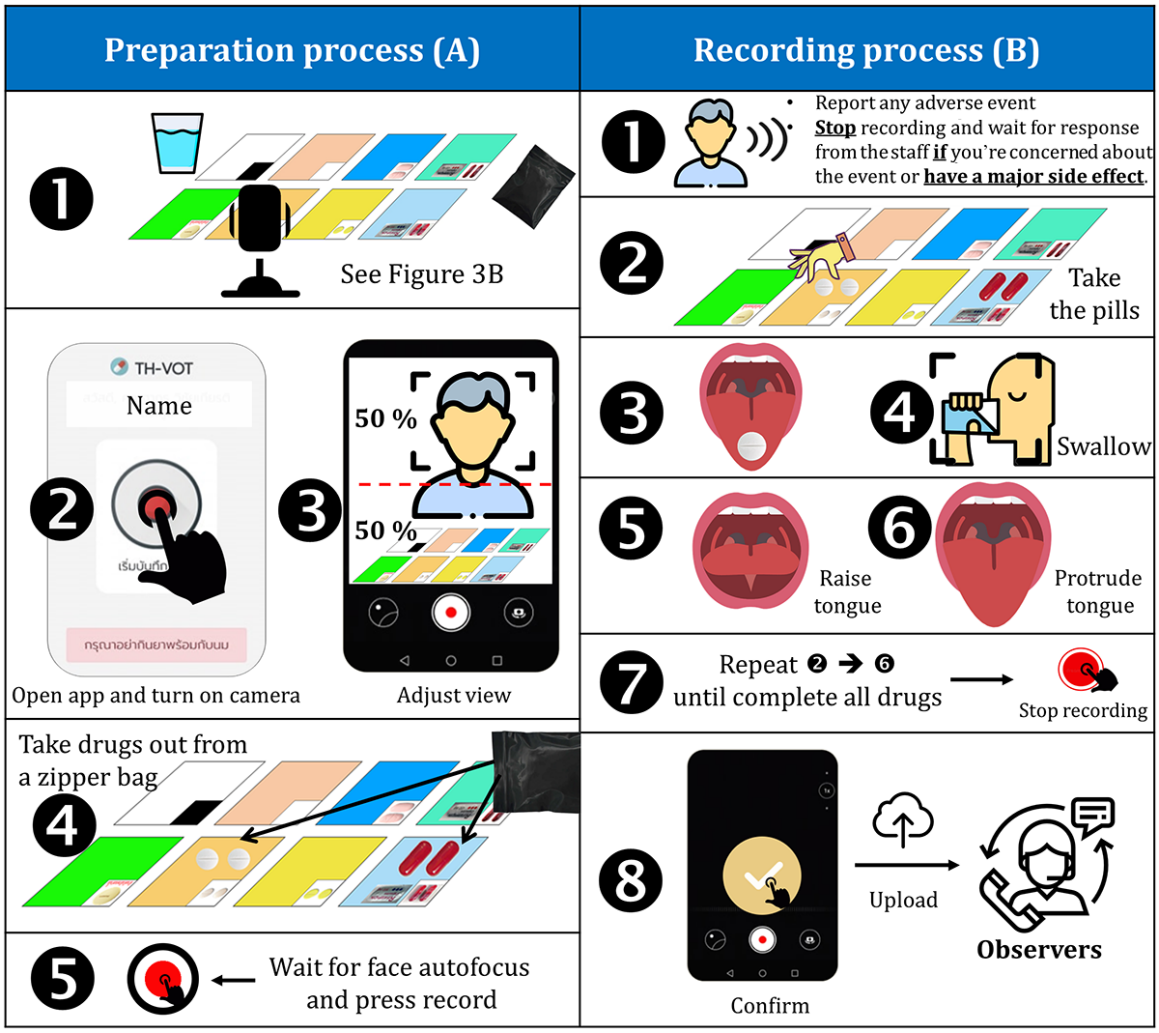
Standard operation procedures of the video-observed therapy at the observers' end.

Instructions for the preparation process for patients with TB (Figure 4A) were as follows:

1. Place the given items on a table as shown in the position guideline (Figure 3B).
2. Open the TH VOT app and turn on the front camera.
3. Adjust the frame as appropriate. The upper half of the frame should contain a full-face image.
4. Remove all the anti-TB drugs from the daily zipper bag and place them on their color labels.
5. Wait for autofocus at the face and press the recording button.

Instructions for the recording process for patients with TB (Figure 4B) were as follows:

1. Depending on the condition:
  If you are feeling unwell owing to the drugs, please report your adverse event through the app, call the TB staff, and temporarily stop taking the pills.
  If you have any major side effects (vision changes, jaundice, confusion, vomiting, and skin rash), stop the medication and call the TB staff.
  If you experience anaphylaxis, call the emergency department of Na Yong Hospital (Tel. +66-075-299-099)
  Otherwise, continue to step 2.
2. Remove the pill from its label and place it on your tongue.
3. Protrude your tongue to show the tablet or capsule.
4. Swallow it. Note that multiple tablets or capsules can be placed on your tongue as convenient before swallowing in order to reduce water intake.
5. Raise your tongue to show your sublingual area.
6. Protrude your tongue to expose your palate area.
7. Repeat steps 2-6 until completion of the daily dose and stop recording.
8. Confirm your video and upload it to the TH VOT database.

#### SOPs of the VOT at the Observers’ End (Observer SOP)

Instructions for the registration process for the observers (Figure 2A) were as follows:

1. Request informed consent from the patient.
2. Provide the items listed in Textbox 1 to the patient.
3. Register the patient on the secure web-based platform and let the patient set his/her password privately.
4. Register the prescription for the patient.
5. Install the TH VOT app on the patient’s smartphone.
6. Let the patient attempt to log into his/her account and use the smartphone to capture a photograph of his/her face and upload it to the system as proof for future facial identification.
7. Train the patient to use the TH VOT app in accordance with the patient SOP.
8. Appoint the time range for the patient’s drug-taking and explain the reminding process.

Instructions for the reviewing process for the observers (Figure 2B) were as follows:

1. In the appointed time range, open the review dashboard of the secure web-based platform, check the rows of video sessions uploaded and consider the following:
  If no video is sent, call the patient to provide him/her a reminder, and then continue to step 8.
  Otherwise, click the eye button in the row with an unapproved status to pop up a window of video verification, and then continue to step 2.
2. Click “Play” to start the video.
3. Confirm the identity of the patient in the video.
4. Listen to any adverse event complaints and check the boxes of common side effects or type in other adverse events found in the video.
5. Identify the tablets or capsules of the anti-TB drugs taken by the patient by using the color board, and enumerate the consumed pills.
6. Use the checklist provided in Figure 4B. Assign 1 score to each correctly performed procedure (ranging 2-7). If there is any mistake, call the patient and provide advice to carry out the correct procedure in future.
7. Click “Save” to close the pop-up window.
8. Contact and respond to the patient if any adverse event or any other issue that occurs within 24 hours.
9. Take notes of any response in the record row of the dashboard.

### Identification of Potential System Improvements

This study was approved by the Human Research Ethics Committee, Faculty of Medicine, Prince of Songkla University (approval# 64-03618-9). The implementation was approved by the research team and the chief medical officer of Na Yong Hospital.

#### Study Design

A mixed-methods pilot study was conducted through users’ experiences of 2-week usage. The potential for system improvement was identified on the basis of two aspects: user compliance and user suggestion.

#### Participants

In Na Yong district, 6 PCUs served a total of 16 patients with active TB. Only 3 patients from 3 different PCUs had a smartphone and were thus recruited in our study. One TB staff as an observer and 1 healthy volunteer as a simulated patient were then recruited to attempt using the TH VOT system in each of these 3 PCUs. All participants were trained to use the TH VOT system in accordance with the aforementioned SOPs.

#### Data Collection

After the 2-week usage, the system’s data records were automatically collected in the database of the TH VOT system since the beginning of the observation period. The video session records in the TH VOT system were retrieved to assess the compliance levels of all participants.

The participants were assigned to score the validated Thai user experience questionnaire (UEQ) [17]. The UEQ contained six dimensional scales: attractiveness (6 items), perspicuity (4 items), efficiency (4 items), dependability (4 items), stimulation (4 items), and novelty (4 items). The items were developed on the basis of the shape of a semantic differential scale with 7-point scales. Two words with opposite meanings represented each item. The order of the itemized terms was randomized with half of the scale’s items beginning with a positive term (+3) and the other half of the items beginning with a negative term (–3). The neutral point was always 0. Cronbach α coefficient values from the original paper were as follows: attractiveness=.86, perspicuity=.71, efficiency=.79, dependability=.69, stimulation=.88, and novelty=.84 [18].

When participants completed scoring, the scores were processed in the researcher’s computer and individually revealed to the score raters, and each dimensional score was compared with the upper limit of the general thresholds [19]. Then, each participant was privately asked to explain why those scores were assigned. The reflections on the users’ experiences and suggestions for further development were collected using unstructured in-depth interviews to clarify users’ reasons and their compliance. Participant interviews lasted 30-45 minutes each.

## Results

### Results Overview

Of the 9 participants initially recruited, 8 completed the study and 1 patient with TB retreated after 1 day of use owing to crashing of the app. Data from the 8 remaining participants were used to identify the potential for system improvement.

### User Compliance

Overall, 70 video sessions were expected (2 from real patients and 3 from simulated patients over 14 days). A total of 60 sessions were recorded and submitted. Of these, 37 sessions were inspected by the staff within 24 hours.

### User Suggestion

UEQ scores were revealed to each participant before starting their in-depth interviews. As shown in Table 1, all patients and simulated patients assigned higher scores in all dimensions than the upper limit of the general thresholds. The observers assigned high scores on attractiveness and novelty but poor scores on other dimensions.

According to the in-depth interviews, the patients and simulated patients forgot to take medication on some days, and the observers did not make a phone call to remind them. The push notification of the app was sometimes not seen as well. The observers requested to have the automatic notification system and suggested the need for auditing by their supervisors.

**Table 1:** The users’ experience scores.

Participant group and #		User Experience Questionnaire score					
		Attractiveness	Perspicuity	Efficiency	Dependability	Stimulation	Novelty
Good benchmark^a^		1.41	1.84	1.43	1.53	1.10	0.87
**Observer**							
	O1	2.17	1.25	0.75	1.50	–0.25	1.00
	O2	1.67	0.25	0.50	1.25	–0.75	1.00
	O3	1.67	0.75	0.75	1.25	–1.00	1.00
**Simulated patient**							
	S1	2.67	2.25	1.75	1.75	1.00	1.00
	S2	1.50	2.00	1.75	2.00	1.50	1.25
	S3	2.17	2.50	1.50	2.00	1.50	1.25
**Real patient**							
	P1	1.67	2.5	1.75	1.75	1.50	1.25
	P2	2.17	2.00	1.50	2.00	1.50	1.00
	P3	N/A^b^	N/A	N/A	N/A	N/A	N/A

^a^Upper limit of the general thresholds [19].

^b^N/A: not available; this patient withdrew from the trial because the app crashed.

## Discussion

### Principal Findings

According to the participant recruitment requirements, possession of a smartphone was the main problem of the A-VOT system, as only 2 patients owned a usable smartphone in this study. However, this problem could be resolved by providing smartphones with public cellular internet to patients who did not have a smartphone or their caretakers. An Android smartphone compatible with the TH VOT system could be purchased for approximately 900-1200 Baht (US $27.44-36.59), which was less than the total cost of drugs for standard TB treatment per patient: 1300-1800 Baht (US $39.63-54.88) [20]. After the patient was cured, the smartphone would be returned to the health service for use by the next patient with TB.

For user compliance, the level of use was not high on the patients’ end and low on the observers’ end compared to those in the United States and the United Kingdom [15,16]. Two more system elements would be needed: the audit and notification systems. The chief of the TB staff should be assigned to audit the quality of the VOT used by the patients and observers. A notification should be set up to provide feedback and notify the evaluation to the patients and observers.

Potential system improvements indicated by all users included the notification system, which was inactivated owing to budget-related problems. A largely well-known notification system, such as SMS or LINE, may be more effective to stimulate users than the current push notification. To overcome this problem, the option of an application programming interface of LINE notifications must be activated in further studies.

### Limitations

This pilot study was limited by its small budget. Our findings indicate the need for adequate financial preparation to cover smartphone lending, audit management, and notification systems. The UEQ scores of this study could not be interpreted to conclusively determine usability levels owing to the small sample size of our study. Two weeks of observation may not be long enough to assess the burnout effect on users. Furthermore, simulated patients who did not take TB medication could not appropriately represent the side effect detection process.

### Conclusions

To improve the TH VOT system, smartphones should be lent to patients with TB who do not own a smartphone. An audit system and web-based notification system to remind the observers and patients must be set up.

## Abbreviations

A-VOT: asynchronous video-observed therapy
DOT: directly observed therapy
PCU: primary care unit
SOP: standard operating procedure
S-VOT: synchronous video-observed therapy
TB: tuberculosis
TH VOT: Thai video-observed therapy
UEQ: user experience questionnaire
VOT: video-observed therapy
